# A Case Report Detailing a Rare Presentation of Idiopathic Intracranial Hypertension With Atypical Symptoms

**DOI:** 10.7759/cureus.59072

**Published:** 2024-04-26

**Authors:** Salima Tibi, Harshitha Gedda, Muhammad Haris, Golda K Joy, Srushti Patil

**Affiliations:** 1 Medicine, Altinbas University, Istanbul, TUR; 2 Medicine, Sri Padmavathi Medical College for Women, Tirupati, IND; 3 Medicine, Wah Medical College, Punjab, PAK; 4 General Practice, St John's Medical College, Bengaluru, IND; 5 Medicine, Smt. Mathurabai Bhausaheb Thorat (SMBT) Sevabhavi Trust Medical College, Nashik, IND

**Keywords:** pseudotumor cerebri syndrome (ptcs), pseudotumor cerebri, headache, intracranial hypertension, idiopathic intracranial hypertension (iih)

## Abstract

Idiopathic intracranial hypertension (IIH), formerly known as pseudotumor cerebri, represents a challenging diagnostic entity in neurology, characterized by elevated intracranial pressure of unknown origin. The classical clinical triad of headache, visual disturbances, and papilledema provides a well-established framework for diagnosis; however, the heterogeneity of IIH presentations, combined with the absence of an overt causative factor, continues to perplex clinicians. This case report delves into the complexities of a rare IIH presentation in a 32-year-old male, highlighting the need for a nuanced understanding of this condition beyond its traditional confines.

## Introduction

Idiopathic intracranial hypertension (IIH) has long been regarded as an intriguing and perplexing neurological disorder, presenting clinicians with a diagnostic puzzle that extends beyond the conventional boundaries of recognized symptoms and patient demographics. Historically labeled as pseudotumor cerebri, IIH challenges our understanding of intracranial dynamics, manifesting as increased pressure within the cranial vault without a clear underlying cause. While the classic triad of headache, visual disturbances, and papilledema serves as a diagnostic anchor, the broader spectrum of IIH presentations continues to evolve, encompassing a diverse array of clinical manifestations [1].

The traditional association of IIH with young, overweight females often directs the diagnostic gaze towards this demographic, potentially leading to oversight when confronted with atypical cases. Our case report unfolds a distinctive narrative, introducing a 32-year-old male patient with an atypical clinical presentation. Beyond the classic symptoms, this individual presented with persistent nausea, dizziness, and intermittent pulsatile tinnitus, prompting an initial exploration of common vestibular and migraine etiologies. The subsequent discovery of bilateral sixth nerve palsy in the neurological examination added a layer of complexity, steering the diagnostic trajectory toward an unexpected conclusion [2].

The evolving understanding of IIH requires a departure from rigid demographic stereotypes, urging clinicians to embrace a broader perspective in their diagnostic approach. Our case not only challenges the conventional age and gender associations but also emphasizes the diverse array of symptoms that can herald IIH. As we navigate through the intricacies of this unique presentation, we aim to contribute to the growing body of knowledge surrounding IIH, advocating for an expanded awareness that transcends classical clinical paradigms. By unraveling the nuances of atypical presentations, we aspire to enhance diagnostic acumen, ultimately improving patient outcomes in cases that deviate from the expected norm. 

## Case presentation

The patient, a previously healthy 32-year-old male, 172 cm tall and 70 kg in weight, presented to our neurology clinic with a three-month history of persistent nausea, intermittent dizziness, and pulsatile tinnitus. These symptoms, seemingly unrelated to traditional IIH presentations, prompted an initial evaluation for common vestibular disorders and migraines. Despite extensive investigations, including audiometry and vestibular function tests, no definitive cause for his symptoms emerged.

Upon a more detailed neurological examination, the discovery of bilateral sixth nerve palsy raised concerns regarding a potential intracranial etiology. There was bilateral abducens nerve palsy, normal vitamin B1 deficiency, no sarcoidosis, no thyroid disease, or no signs of myasthenia gravis.

Upon fundus examination, the patient presented with bilateral papilledema. The optic discs appeared swollen, with blurred margins and hyperemia. Additionally, there were signs of peripapillary hemorrhages and cotton wool spots in both eyes, indicative of retinal nerve fiber layer ischemia. Venous engorgement and tortuosity were also observed, suggesting elevated intracranial pressure affecting the retinal vasculature.

Furthermore, there was evidence of optic disc leakage on fluorescein angiography, indicating compromised blood-retinal barrier integrity due to increased intracranial pressure. These findings were consistent with the diagnosis of idiopathic intracranial hypertension (IIH) and correlated with the clinical presentation of persistent nausea, intermittent dizziness, and pulsatile tinnitus, along with bilateral sixth nerve palsy.

This unexpected finding prompted a comprehensive workup, including a lumbar puncture to assess cerebrospinal fluid (CSF) dynamics. Analysis of the CSF revealed an elevated opening pressure exceeding 300 mm H2O, confirming the diagnosis of IIH. Notably, the patient's body mass index (BMI) was within the normal range, challenging the conventional association of IIH with obesity.

**Figure 1:**
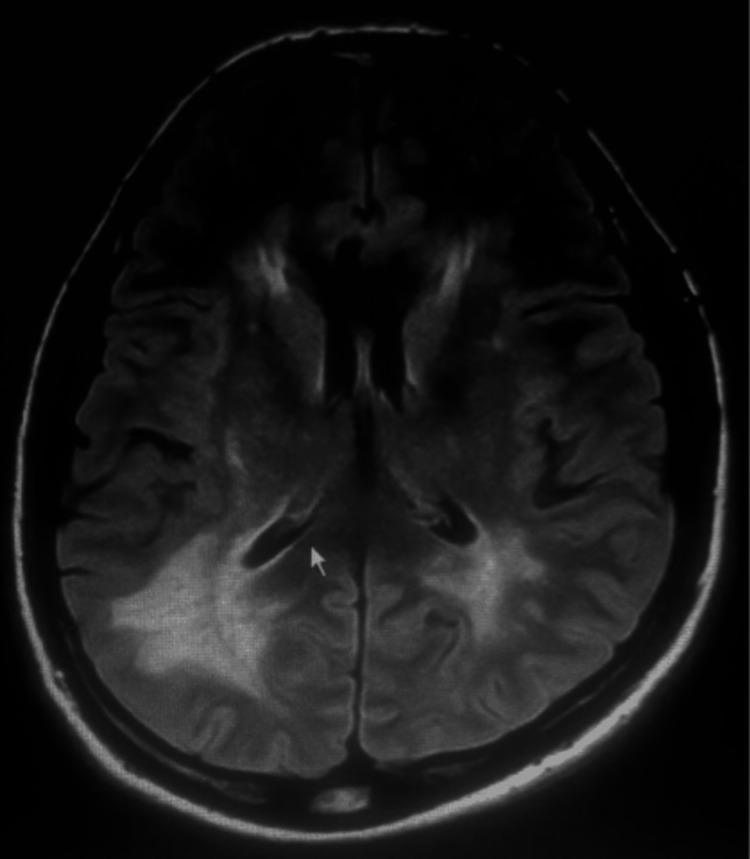
MRI indicating idiopathic intracranial hypertension

Neuroimaging, including magnetic resonance imaging (MRI) and magnetic resonance venography (MRV), was performed to rule out structural abnormalities and venous sinus thrombosis. The imaging studies showed no evidence of mass lesions, hydrocephalus, or venous abnormalities, further emphasizing the idiopathic nature of the intracranial hypertension (Figure 1).

The patient was initiated on acetazolamide therapy for intracranial pressure management, along with counseling on lifestyle modifications. Regular follow-ups revealed a gradual resolution of his presenting symptoms and improvement in the sixth nerve palsy. Serial lumbar punctures confirmed a sustained reduction in CSF pressure, supporting the efficacy of the treatment regimen.

## Discussion

The atypical presentation of idiopathic intracranial hypertension (IIH) in this case prompts a deeper exploration of the diagnostic challenges associated with this complex disorder. While IIH is traditionally linked to a demographic profile of young, overweight females, the presented case challenges these stereotypes, highlighting the need for clinicians to maintain a broad differential diagnosis, even when faced with symptoms seemingly unrelated to the classical triad of headache, visual disturbances, and papilledema [1].

One noteworthy aspect of this case is the initial manifestation of symptoms, including persistent nausea, intermittent dizziness, and pulsatile tinnitus. These symptoms, though not conventionally associated with IIH, played a pivotal role in steering the diagnostic process. The atypical nature of the presentation underscores the importance of recognizing the heterogeneity of IIH, which may present with a myriad of clinical features that extend beyond the established norms [3].

The bilateral sixth nerve palsy identified during the neurological examination further contributed to the diagnostic puzzle. Over 10% of IIH can show sixth nerve palsy. The identification of these palsies prompted a comprehensive diagnostic workup, leading to the confirmation of elevated intracranial pressure through lumbar puncture, a key diagnostic step in IIH evaluation [1].

Interestingly, our patient did not exhibit the characteristic association of IIH with obesity. This challenges the conventional understanding of IIH pathophysiology and emphasizes the need for a nuanced approach to patient evaluation. While obesity remains a significant risk factor for IIH, our case suggests that clinicians should remain vigilant in considering IIH across a broader spectrum of patient demographics and body habitus [2].

Neuroimaging, including magnetic resonance imaging (MRI) and magnetic resonance venography (MRV), revealed no structural abnormalities or venous sinus thrombosis, supporting the diagnosis of idiopathic intracranial hypertension. This aligns with the notion that IIH is a diagnosis of exclusion, requiring the thorough elimination of other potential causes of elevated intracranial pressure [4].

The management of our patient included the initiation of acetazolamide therapy for intracranial pressure reduction and lifestyle modifications. The typical starting dose of acetazolamide for idiopathic intracranial hypertension (IIH) is 500 mg orally twice daily. The gradual resolution of symptoms, improvement in sixth nerve palsy, and sustained reduction in cerebrospinal fluid pressure demonstrated the effectiveness of the treatment strategy. Long-term follow-up is crucial to monitor for symptom recurrence and to adjust the management plan accordingly [5].

This case contributes to the ongoing discourse on IIH by broadening the understanding of its clinical spectrum. It underscores the importance of a comprehensive and individualized diagnostic approach that considers atypical presentations, guiding clinicians to navigate beyond established paradigms. Moreover, this case highlights the need for heightened awareness among healthcare professionals to ensure timely diagnosis and appropriate management, particularly in cases that deviate from the expected demographic and clinical norms associated with IIH.

## Conclusions

Idiopathic intracranial hypertension can manifest with atypical symptoms, making diagnosis challenging. Clinicians should maintain a high index of suspicion, especially in cases with unusual presentations, to ensure timely intervention and prevent potential complications. This case contributes to the evolving understanding of IIH, emphasizing the importance of a comprehensive diagnostic approach and tailored management strategies. By unraveling the intricacies of atypical presentations, we aim to enhance the diagnostic acumen of clinicians, ultimately improving patient outcomes in cases that deviate from the expected norm.
